# High Expression of *PPM1D* Induces Tumors Phenotypically Similar to *TP53* Loss-of-Function Mutations in Mice

**DOI:** 10.3390/cancers13215493

**Published:** 2021-10-31

**Authors:** Jelena Milosevic, Susanne Fransson, Miklos Gulyas, Thale K. Olsen, Gabriel Gallo-Oller, Diana Treis, Lotta H. M. Elfman, Margareta Wilhelm, Tommy Martinsson, Ninib Baryawno, Per Kogner, John Inge Johnsen

**Affiliations:** 1 Childhood Cancer Research Unit, Department of Women’s and Children’s Health, Karolinska Institutet, 17177 Stockholm, Sweden; thale.kristin.olsen@ki.se (T.K.O.); ggallo@alumni.unav.es (G.G.-O.); diana.treis@ki.se (D.T.); lotta.elfman@ki.se (L.H.M.E.); n.baryawno@ki.se (N.B.); per.kogner@ki.se (P.K.); 2 Center for Regenerative Medicine, Massachusetts General Hospital, Boston, MA 02114, USA; 3 Department of Laboratory Medicine, Institute of Biomedicine, University of Gothenburg, 41345 Gothenburg, Sweden; susanne.fransson@clingen.gu.se (S.F.); tommy.martinsson@clingen.gu.se (T.M.); 4 Department of Immunology, Genetics and Pathology, Uppsala University, 75185 Uppsala, Sweden; miklos.gulyas@igp.uu.se; 5 Department of Microbiology, Cell and Tumor Biology, Karolinska Institutet, 17177 Stockholm, Sweden; Margareta.Wilhelm@ki.se

**Keywords:** *PPM1D*, p53, genetically engineered mouse model, adenocarcinoma, lymphoma, neuroblastoma

## Abstract

**Simple Summary:**

Aberrant expression of the *PPM1D* gene which encodes a phosphatase called WIP1 is frequently observed in cancers of different origins. WIP1 is a negative regulator of the tumor suppressor p53. Improper inactivation of p53 results in genomic instability and can induce neoplastic transformation. We show that overexpression of *PPM1D* induces tumors in mice similar to cancers harboring p53 mutations. Our results suggest that *PPM1D* can act as an oncogenic driver by inducing genomic instability, impaired growth arrest, and apoptotic escape that can result in neoplastic transformation and malignant tumor development.

**Abstract:**

*PPM1D* is a negative regulator of p53 and genomic aberrations resulting in increased activity of *PPM1D* have been observed in cancers of different origins, indicating that *PPM1D* has oncogenic properties. We established a transgenic mouse model overexpressing *PPM1D* and showed that these mice developed a wide variety of cancers. *PPM1D*-expressing mice developed tumors phenotypically and genetically similar to tumors in mice with dysfunctional p53. T-cell lymphoblastic lymphoma was the most frequent cancer observed in these mice (55%) followed by adenocarcinomas (24%), leukemia (12%) and other solid tumors including neuroblastoma. Characterization of T-cell lymphomas in mice overexpressing *PPM1D* demonstrates *Pten*-deletion and p53-accumulation similar to mice with p53 loss-of-function. Also, *Notch1* mutations which are recurrently observed in T-cell acute lymphoblastic lymphoma (T-ALL) were frequently detected in *PPM1D-*transgenic mice. Hence, *PPM1D* acts as an oncogenic driver in connection with cellular stress, suggesting that the *PPM1D* gene status and expression levels should be investigated in *TP53* wild-type tumors.

## 1. Introduction

The protein phosphatase magnesium-dependent 1 delta (*PPM1D*) gene encodes a nuclear serine/threonine phosphatase included in the PP2C family of phosphatases named WIP1 (wild-type p53-induced phosphatase 1) [1]. WIP1 is a critical regulator of DNA damage response and cell-cycle progression by its ability to regulate the activity of the tumor-suppressor protein p53, ATM, CHK1/2, and other key molecules involved in cell-cycle progression, DNA repair mechanisms, and apoptosis [2,3,4,5,6,7]. In concordance, *PPM1D* mutations, amplifications, gene fusions, and WIP1 overexpression have been observed in various cancers [8,9,10,11,12,13,14,15,16,17].

Inactivation of p53 is seen in more than 50% of human cancers and results in genomic instability, impaired growth arrest, and apoptotic escape allowing for continued cell proliferation and neoplastic induction [18,19]. p53 is induced and stabilized following DNA damage through multiple posttranslational modifications, ultimately leading to transcriptional activation of p53-target genes orchestrating cell-cycle progression, DNA repair mechanisms, and apoptosis [18]. In order to facilitate a rapid switch between an active and inactive state of p53, several mechanisms regulating the activity of p53 have been described. One important factor is the *MDM2* oncogene encoding an E3 ubiquitin ligase [20,21]. MDM2 expression is induced by p53 which results in p53–MDM2 complex formation, MDM2-mediated ubiquitination, and proteasomal degradation of p53 [21]. This p53–MDM2 autoregulatory loop is controlled by ATM and ATR as well as other kinases that, immediately following DNA damage, phosphorylate MDM2 and p53 in a manner that inhibits the p53–MDM2 interaction leading to stabilization of p53 [22,23,24]. In order to restore the normal homeostatic state of the cell after completion of DNA repair, a set of phosphatases dephosphorylate p53 and MDM2 to allow for p53–MDM2 interaction and proteasomal degradation of p53. The expression level of WIP1 is controlled directly by p53 through binding to regulatory elements located in the 5′-untranslated region of the *PPM1D* gene [1,25]. WIP1 has been shown to dephosphorylate and inactivate p53 (Ser15) as well as the p53-activating kinases ATM, CHK1, and CHK2 that phosphorylate p53 at Ser15 and Ser20, respectively [2,3,4,5,6,7]. WIP1 also dephosphorylates MDM2 at Ser395, a site phosphorylated by ATM [26]. The dephosphorylation of p53 and p53-regulating kinases allows MDM2 to interact with p53 and mediate proteasomal degradation [26]. WIP1 inactivation or depletion in tumor cells with high expression of WIP1 results in reduced cell viability in vitro or suppressed tumor growth in preclinical rodent models [6,27,28,29,30,31]. This is supported by the demonstration that *Ppm1d*-deficient mice show a delayed onset of mammary gland tumor development whereas an accelerated onset of tumors is evident in mice overexpressing *PPM1D* and *ERBB2* in the mammary gland as well as reduced survival of *APC^min^* mice developing colon cancer [27,32,33]. Taken together, this suggests that WIP1 has important functions during tumorigenesis and that *PPM1D* has properties of an oncogene although no direct evidence for its being an oncogenic driver has been reported so far.

In an attempt to investigate the potential role of WIP1 during tumorigenesis we developed a transgenic mouse model overexpressing *PPM1D.* We show that this overexpression results in the development of cancers phenotypically similar to tumors arising in mice with dysfunctional p53 when subjected to low-dose irradiation. 

## 2. Results

### 2.1. Mice Carrying the PPM1D Transgene Develop Tumors in Response to Cellular Stress 

**Figure 1:**
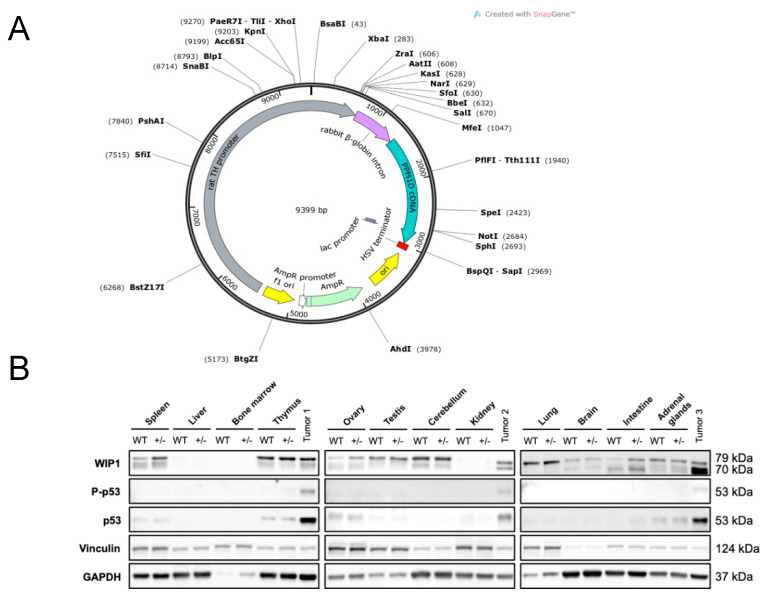
*PPM1D*-transgenic mice show increased WIP1 expression and develop widespread tumors with wild-type p53 accumulation. (**A**) Transgene construct. Human *PPM1D* cDNA (blue) *PPM1D,* ligated downstream of the rat tyrosine hydroxylase promoter (gray) and rabbit β-globin enhancer (violet). Herpes simplex virus (HSV) thymidine kinase gene sequence was used as a transcription terminator (red). (**B**) Thymic lymphoma shows increased WIP1 protein expression, p53^Ser15^ phosphorylation, and accumulation of total p53. Protein expression of WIP1 was analyzed in wild-type and *PPM1D* heterozygous (+/−) mouse tissues. Three different mouse thymic lymphoma tumor samples were also included (tumors 1–3). Vinculin and GAPDH protein levels were used as loading controls.

To characterize the role of *PPM1D* in tumor development, we constructed genetically engineered C57BL/6N mice overexpressing WIP1 through pronuclear injection of the human *PPM1D* gene controlled by the rat tyrosine hydroxylase (TH) promoter (Figure 1A). Three founder lines were generated with intact transmission of the *PPM1D* transgene to following generations. 

**Figure 2:**
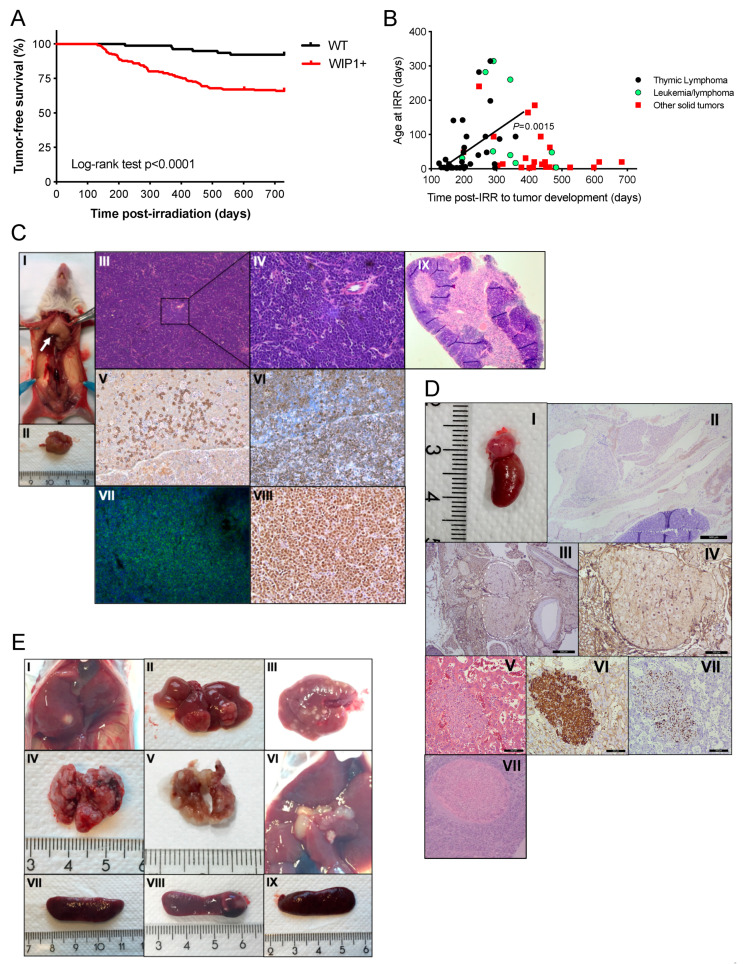
*PPM1D*/WIP1-transgenic mice have increased carcinogenic susceptibility compared to wild-type mice following irradiation. (**A**) Kaplan–Meier analysis of irradiated *PPM1D*/WIP1-transgenic mice with the endpoint defined as detection of tumors. Mice were either homozygous or heterozygous for the human *PPM1D* gene. WIP1-positive transgenic mice (n = 210) and their wild-type littermates (*n* = 79) with different degrees of 129 × 1/SvJ-strain background were subjected to one 4.5 Gy sublethal whole-body irradiation dose at different days of age (1–314 days old). Mice that were positive for the human *PPM1D* gene frequently developed more tumors compared to wild-type mice (Log rank test, *p* < 0.0001) following irradiation. Among the *PPM1D*/WIP1-positive mice, 69 mice developed tumors compared with six mice in the wild-type group (Fisher’s exact test, *p* < 0.0001). The odds ratio of developing cancer in the *PPM1D*/WIP1-positive group compared to the wild-type group was 6.3 (95% confidence interval 2.7–14.2). (**B**) Time to tumor development of thymic lymphomas post-irradiation (IRR) correlated positively with age at irradiation (Pearson, *p =* 0.0015. Spearman, *p* = 0.001); the majority of lymphomas manifested < 300 days. There was no correlation between age at irradiation and time to tumor development in mice diagnosed with other solid tumors (Pearson, *p* = 0.0658, Spearman, *p* = 0.1589) and mice diagnosed having leukemia/lymphoma (Pearson, *p* = 0.3651, Spearman, *p* = 0.1977). (**C**) Thymic lymphomas were the most common disease manifestation in irradiated human *PPM1D*/WIP1-positive mice. Representative image of thoracic tumor in situ indicated by the arrow (I) and after dissection (II). Microscopy at 4× (III) and 20× (IV) magnification after hematoxylin and eosin (H&E) staining showed atypical lymphoblastic cells having intense mitotic activity. (V) Infiltration of B220-positive cells (20× magnification). (VI–VII) The majority of cells are positive for the pan-T cell marker CD3 (20×magnification). (VIII) Staining for Ki-67 was highly positive, indicating high proliferative activity (20× magnification). The thoracic organs, mediastinum (heart base and hilum of the lung), thymus tumor, and lymph nodes displayed extensive infiltrates of neoplastic and highly malignant lymphoblasts consistent with lymphoblastic lymphoma. (IX) H&E staining of a normal mouse thymus (4× magnification). (**D**) Adrenal neuroblastic tumor and liver metastasis from a *PPM1D*/WIP1 transgenic mouse. (I) Macroscopic view of the adrenal tumor. (II) Hematoxylin/eosin staining of the adrenal tumor. (III–IV) PHOX2B-staining was positive, indicating neural crest origin of this neuroblastoma-like tumor. (V–VII) Liver metastasis with hematoxylin/eosin staining, synaptophysin staining, and PHOX2B staining, respectively. (VII) H&E staining of a normal mouse adrenal showing both the medulla and the cortex (4× magnification). (**E**) *PPM1D*-transgenic mice exhibit widespread tumor malignancies. Representative macroscopic photographs: tumor mass in the liver (I), multiple hepatic metastases (II,III), lung carcinosis/metastases (IV,V), enlarged lymph nodes (VI), and splenomegaly (VII–IX) indicative of high tumor burden.

Elevated WIP1 protein expression was detected in the spleen, ovary, and intestine in mice hemizygous for *PPM1D* compared to wild-type mice (Figure 1B, File S1). However, no increase in tumor development was observed in these *PPM1D-*transgenic mice. We therefore backcrossed the C57BL/6N *PPM1D* +/− mice for eight generations with 129 × 1/SvJ mice, which are more prone to tumor formation, in order to establish a near 100% 129 × 1/SvJ background. In addition, given the wide-ranging actions of WIP1 during DNA damage repair, we hypothesized that overexpression of WIP under cellular stress could have the potential to induce neoplasms arising in response to extrinsic cellular DNA damage. Hence, *PPM1D*-overexpressing animals and their wild-type littermates were exposed to 4.5 Gy of sub-lethal whole-body irradiation at different ages (1–314 days old). Irradiated transgenic mice carrying the *PPM1D* gene had significantly higher tumor incidence (34% tumor probability) compared to wild-type mice (7.8%; *p* < 0.0001) 100–300 days post-irradiation (Figure 2A). 

The majority of cancers detected in *PPM1D*-transgenic mice were similar to those reported in irradiated p53-mutant mice post-irradiation [32,33,34], indicating a similar tumor phenotype caused by p53 impairment rather than p53 absence. Thymic lymphoblastic lymphoma was the most frequent malignancy observed (*n* = 41; Figure 2B,C and Table 1), compared to four thymic lymphomas detected in irradiated wild-type mice (*p* < 0.001). Other malignancies manifested in *PPM1D*-transgenic mice were leukemias/lymphomas (*n* = 9), different adenocarcinomas/adenomas (*n* = 18), and sarcomas (*n* = 4) (Table 1). Additionally, we also identified mice with multiple primary tumors, one mouse with an adenocarcinoma in the lacrimal gland and an osteosarcoma with lung metastasis, one mouse with T-cell lymphoma, ovary carcinoma, and lung adenocarcinoma, two mice with neuroblastoma, one of which also had liver metastasis (Figure 2D), and a mouse with a gastric adenocarcinoma and leukemia (Table 1). Other tumor manifestations observed in several *PPM1D*-positive mice were spleen, liver, lung, and lymph node metastases (Figure 2E and Table 1). The odds ratio of developing cancer in mice with the *PPM1D* transgene was 6.3 (95% confidence interval 2.7–14.2) compared to wild-type mice.

Comparison of age at irradiation and time to tumor development disclosed a positive correlation, showing that younger *PPM1D*-positive animals were more susceptible to developing thymic lymphomas in response to DNA damage (*p* < 0.01), the majority (38 out of 41) of which arose <365 days post-irradiation (Figure 2B). Protein expression of thymic lymphomas showed high WIP1 expression and increased phosphorylation of p53 compared to non-irradiated thymus from *PPM1D*-positive and wild-type mice (Figure 1B). 

### 2.2. PPM1D-Derived Mouse Lymphoma Tumors Show Frequent Pten and Notch1 Mutations and Activation of Notch Signaling

**Figure 3:**
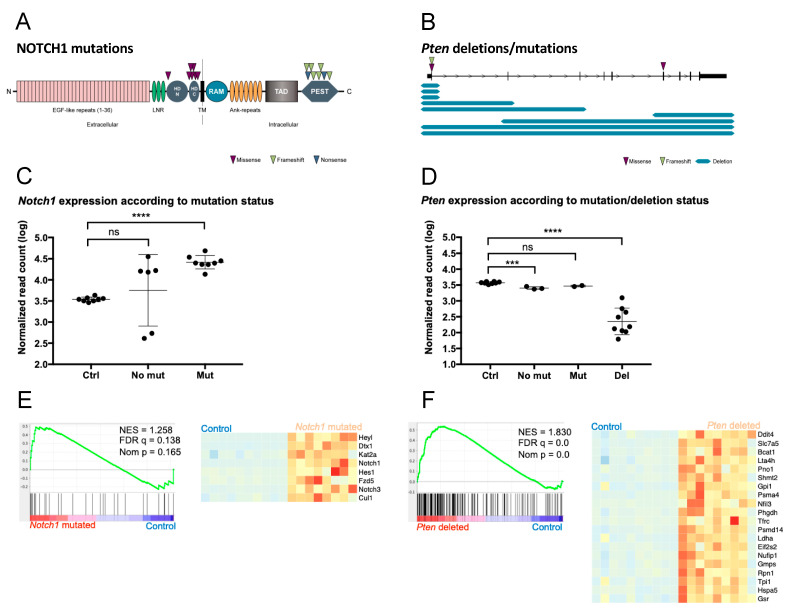
*PPM1D*-induced thymic lymphomas show typical *Notch1* and *Pten* aberrations. (**A**) Schematic overview of the NOTCH1 protein showing the distribution of mutations in 15 whole-exome sequenced lymphomas. Purple triangles indicate missense mutations in the intracellular heterodimerization (HD) domain, whereas green and blue triangles indicate frameshift or nonsense mutations in the extracellular PEST domain. LNR, Lin/NOTCH repeats; HD, heterodimerization domain; TM, transmembrane domain; RAM, RBP-JK-associated molecule region; TAD, transactivation domain; PEST, sequence rich in proline, glutamic acid, serine, and threonine. (**B**) Diagram of the *Pten* gene showing the size and distribution of *Pten* deletions/mutations in lymphomas. The locations of the point mutations are shown with purple and green triangles above the gene diagram. The blue lines below the gene diagram show the size of the deletions identified by exome sequencing. (**C**) Expression of the *Notch1* transcript in controls and tumors with and without *Notch1* mutation. The y axis shows the log value of the number of RNA-seq reads mapping to the *Notch1* gene. The difference between controls and tumors without *Notch1* mutations is not significant (ns), whereas *Notch1* expression is significantly higher in the mutated tumors (adjusted *p* value < 0.0001; Bonferroni multiple comparisons test). **** *p* < 0.0001. (**D**) Expression of *Pten* in controls and tumors with and without *Pten* mutation/deletion. Expression is significantly lower in tumors harboring deletions than in controls (adjusted *p* value 0.0001, Bonferroni multiple comparisons test). *** *p* < 0.001, **** *p* < 0.0001. (**E**) Gene set enrichment analysis comparing *Notch1* mutated tumors to controls demonstrated a significant upregulation of genes related to Notch signaling (MSigDB ”Hallmarks” gene set). Left panel: Enrichment plot from GSEA. Right panel: Heatmap showing the expression (z scores) of core enriched genes. Red color indicates positive z scores; blue color indicates negative z scores. (**F**) Gene set enrichment analysis comparing *Pten* deleted tumors to controls demonstrated upregulation of genes related to the Mtorc1 pathway (MSigDB “Hallmarks” gene set). Left panel: Enrichment plot from GSEA. Right panel: Heatmap showing the expression (z scores) of the top 20 core enriched genes. Red color indicates positive z scores; blue color indicates negative z scores.

To further characterize the tumors that developed in *PPM1D*-transgenic mice, we performed whole-exome sequencing on *PPM1D*-derived T-cell lymphomas. We detected an average of 18 (range 9–34) somatic protein-changing mutations compared to non-irradiated wild-type control mice. Thymic tissues from the two control groups, irradiated wild-type mice, and irradiated *PPM1D*-positive mice without tumors, showed an average of two (range 1–4) and three (range 1–5) somatic protein-changing mutation variants, respectively, similar to non-irradiated non-tumorigenic wild-type mice (Table S1). These data indicate that irradiation alone was not responsible for the accumulation of DNA mutations observed in the tumors. Among the thymic lymphomas, we found *Pten* and *Notch1* to be recurrently altered (Figure 3). In total, 15 activating *Notch1* mutations were detected in 9 of 15 lymphomas (60%) and among these, five tumors had more than one mutation of the *Notch1* gene. Similar to human cancers, the *Notch1* missense variants were found to cluster in the heterodimerization domain (HD) while frameshift or nonsense variants were clustered to the proline–glutamic acid–serine–threonine (PEST) domain (Figure 3A). Of the 15 sequenced lymphomas, *Pten* aberrations were detected in 12 cases (80%) consisting of homozygous deletions (nine cases), missense variants (two cases), and frameshift insertion (one case) (Figure 3B). The three missense and frameshift variants all showed a high level variant allele fraction (variant allele fraction 85–97%) suggesting a near homozygous presence. Mutations of *Pten* and *Notch1* were co-occurring in seven cases (46%) (Table S1). In addition to *Notch1* and *Pten* mutations, aberrations in genes previously associated to human cancers were also observed in *Ikzf1*, *Kras*, *Rac2*, *Trp53*, *Ctnnb1*, *Gli3*, *Zfp36l2*, *Mpdz*, *Dd3x*, *Foxp1*, *Pin1,* and *Ezh2* (Table S1). 

We also performed RNA-sequencing on the tumors and analyzed them using principal component analysis (PCA) and unsupervised hierarchical clustering based on the 200 most variable genes (Figure S1A,B). Differential gene expression analysis (Table S2) identified 4138 upregulated and 4378 downregulated genes in tumors when compared to controls. Tumors harboring *Notch1* mutations had significantly higher expression of the *Notch1* gene compared to controls (Figure 3C). Also, as expected, *Pten* expression was significantly lower in tumors harboring deletions of the gene (Figure 3D). Likewise, gene set enrichment analysis (GSEA) showed corresponding increased expression of genes involved in Notch- and Mtorc1-signalling in *Notch1-* or *Pten-*mutated tumors compared to controls (Figure 3E,F). Thus, tumor development was phenotypically and genetically similar to tumors from p53-impaired mice exhibiting *Pten* deletions and wild-type p53-accumulation [35,36,37] and to human thymic lymphomas which frequently harbor *NOTCH* mutations [35].

## 3. Discussion

Aberrant expression of *PPM1D* caused by chromosomal gains, gene amplification, or activating mutations has been described in multiple cancers and high expression of *PPM1D* often correlates with poor patient prognosis [38]. Moreover, high expression and/or genetic aberration of *PPM1D* is frequently found in cancers with wt*TRP53* suggesting that high protein levels or stability of WIP1 inhibit the activity of p53 that can results in neoplastic transformation and malignant tumor formation [36,37]. For these reasons, multiple inhibitors targeting the anti-tumorigenic activity of PPM1D have been developed [39]. Although some of these inhibitors have proven to be effective in inhibiting tumor cell growth both in vitro and in vivo, none of the inhibitors have been subjected to clinical trial due to poor solubility or substandard pharmacokinetics [39]. The demonstration that *Ppm1d*-deficient mice significantly delayed the formation of ERBB2-induced mammary tumors and that *Ppm1d-*null embryonic mouse fibroblasts were resistant to transformation by RAS, ERBB2, and c-MYC [27] encouraged us to establish a genetically modified mouse model overexpressing WIP1. *PPM1D-*transgenic mice developed tumors of different origin after low-dose irradiation compared to wild-type control mice. The spectrum of observed tumors was highly similar to tumors observed in p53-deficient mice or p53-deficient mice subjected to irradiation [32,33,34,40]. Interestingly, the tumors which developed in our *PPM1D-*transgenic mice are phenotypical similar to mice with *Trp53* mutations but not similar to *Trp53* knockout mice. Mice with *Trp53* deletions mainly develop lymphomas and less frequently sarcomas [33,40] whereas *Trp53* mutant mice, in addition to lymphomas and more frequently sarcoma, also develop carcinomas [34]. This is similar to the spectrum of tumors observed in our *PPM1D-*transgenic mice. Thymic lymphoma derived from *PPM1D-*transgenic mice expresses high levels of wild-type p53 and WIP1 similar to *Trp53* mutated mice showing high levels of mutated p53 [41]. Several human cancers exhibit *PPM1D* gene amplification and/or overexpression of the WIP1 protein. The majority of these tumors also retain wild-type p53 but the functionality is compromised [8,14]. We subjected a group of our mice to low-dose irradiation that induces cellular stress and DNA damage and activates p53. Activation of p53 promotes enhanced expression of endogenous *PPM1D* and WIP1 will dephosphorylate serine and threonine residues on key proteins involved in the DNA damage response, including pSer15 on p53. Increased expression of WIP1 will also stabilize MDM2 and MDMX by dephosphorylation of pSER395 and pSer403, respectively [39]. Enhanced MDM2/MDMX stability target p53 for MDM2-mediated ubiquitination and proteasomal degradation. Together this suggests that high levels of WIP1 protein compromise p53 activity in these cancers.

In addition, both the genomic landscape and expression profiles obtained from DNA and RNA sequencing of PPM1D-induced tumors had similar features with regard to DNA mutations and expression profiles as observed in tumors deriving from p53-impaired mice [42]. The majority of sequenced thymic lymphomas detected in PPM1D-transgenic mice contained deletions in the tumor-suppressor gene *Pten*. In p53^-/-^ mice developing thymic lymphomas, deletion of Pten is an early event occurring in a stem or early T-cell lineage progenitor before rearrangements of the T-cell receptors [42]. We also detected frequent activating *Notch1* mutations in lymphomas from *PPM1D*-transgenic mice. Activating *NOTCH1* mutations is one of the most frequent gene aberration found in human T-cell acute leukemia/lymphoma and the majority of these mutations are located in the PEST or heterodimerization domain [35] similar to the *Notch1* mutations detected in lymphomas from *PPM1D*-transgenic mice. In contrast, lymphomas from p53^-/-^ mice do not exhibit *Notch1* mutation but instead frequently contain activating mutation of *Ikaros* resulting in constitutive activation of the Notch signaling pathway [42]. 

Other studies have shown that overexpression of *PPM1D* together with oncogenes augmented tumor formation in mice or resulted in reduced the survival of mice deficient of the APC tumor-suppressor gene [3,43]. Our results show that *PPM1D* overexpression results in tumor development in mice subjected to DNA stresses. Together this strongly suggests that *PPM1D* is an oncogenic driver through its ability to repress p53 activity that leads to deviant cell cycle arrest, DNA repair, apoptosis, and tumor development.

## 4. Materials and Methods

### 4.1. Ethical Permits

The animal experiments were approved by the regional ethics committee for animal research in Northern Stockholm, appointed and under the control of the Swedish Board of Agriculture and the Swedish Court. All animal experiments were in accordance with national regulations (SFS 1988:534, SFS 1988:539, and SFS 1988:541). For specific approval numbers, please refer to the sections below.

### 4.2. WIP1-Overexpressing Mouse Model

Transgenic WIP1-overexpressing mice were generated by pronuclear injection with random integration of a *PPM1D-*transgenic vector construct, carried out at Karolinska Center for Transgene Technologies (KCTT) and granted according to ethical approval numbers N251-12 and N42-14. 

The construct consisted of rat tyrosine hydroxylase (TH) promoter, the rabbit beta-globin intron to enhance expression, and cDNA for the human *PPM1D* gene (MGC Human *PPM1D* Sequence-Verified cDNA, Clone Id: 5167004, Dharmacon). Herpes simplex virus (HSV) thymidine kinase gene sequence was used as a transcription terminator (Figure 1A). The plasmid was kindly provided by Prof. William Weiss (University of California, San Francisco, CA, USA).

The linearized and purified construct was diluted to 1.5 ng/ul in microinjection buffer (10 mM Tris-HCl, pH7.4, 0.1 mM EDTA) and injected into the pronucleus of C57BL/6NCrl zygotes using a Nikon TE200 microinjection system with Narishige NT-88NEN micromanipulators and a Warner Instruments PLI-100A pico-liter injector. The microinjected embryos were transferred into the oviducts of pseudopregnant Crl:CD1(ICR) female mice using standard surgery techniques and ear biopsies from the resulting 55 offspring were screened for the presence of the *PPM1D* transgene by PCR using forward primer: 5′-CTGGTCATCATCCTGCCTTTCT-3′ and reverse primer: 5′-GCCTTTCCCCGAGACTTCG-3′ (Sigma-Aldrich, St. Louis, MO, USA). Six transgenic animals were found of which four founder animals were further established based on their ability to pass on the human *PPM1D*-transgene to their offspring.

In order to achieve a more tumor-permissive genetic background, these four transgenic mouse-lines were backcrossed with 129X1/SvJ mice, aiming for ten generations of backcrossing which should result in approximately 100% 129X1/SvJ background. Backcrossing was carried out in accordance with ethical permit number N641-12. Throughout breeding, mice were monitored closely for development of palpable abdominal tumors or other disease manifestations up to 548 days (1.5 years) of age.

### 4.3. Irradiation of Mice

Using a linear accelerator (X-RAD 320, Biological Irradiator, North Branford, CT, USA) with a dose rate of 0.95 Gy/min at 320 KV and a radiation field of 20 × 20 cm, PPM1D-transgenic mice from three established transgenic lines and their wild-type littermates resulting from heterozygous breeding pairs with different degrees of 129X1/SvJ strain background (three to seven generations of backcrossing from C57BL/6N to 129X1/SvJ background) were subjected to whole-body irradiation at a single sublethal dose of 4.5 Gy (ethical approval N290-15), after which they were monitored daily in their usual pathogen-free environment. Mice were exposed to irradiation at different ages (1 to 314 days old). Littermates were always irradiated simultaneously. The mice were followed up to 548 days (1.5 years) of age.

### 4.4. Immunoblotting

Protein extraction, determination of protein content, SDS-PAGE under reducing conditions, electroblot, and immunoreaction detection were carried out as previously described [44]. Briefly, total proteins were extracted using RIPA buffer (Thermo Fisher Scientific, Waltham, MA, USA) supplemented with Halt™ Protease and Phosphatase Inhibitor Cocktail (Thermo Fisher Scientific) and 50 ug of total protein were resolved by reducing SDS-PAGE and transferred onto a nitrocellulose membrane. The membranes were blocked with 5% non-fat milk in TBS-T bufferand incubated with anti-WIP1, anti-phospho-p53, and anti-p53, all from Cell Signaling, diluted 1:1000 at 4 °C overnight. Anti-Vinculin and anti-GADPH from Abcam were diluted 1:4000 and 1:10,000, respectively, at room temperature for 1 h. Chemiluminescence visualization of antibodies was performed with Amersham^TM^ ECL^TM^ Prime Western Blotting Detection Reagent (GE Healthcare). Visualization and imaging of signal was performed with ImageQuant LAS 4000 (GE Healthcare).

### 4.5. Immunohistochemistry of Transgenic Mouse Tissue Samples

Formalin-fixed and paraffin-embedded transgenic mouse tissue sections were deparaffinized in xylene and graded alcohols, hydrated, and washed in phosphate-buffered saline (PBS). After antigen retrieval in sodium citrate buffer (pH 6) in a microwave oven, endogenous peroxidase activity was blocked by 0.3% H_2_O_2_ for 15 min. Biotin blocking was preformed using an avidin/biotin blocking kit (Vector Laboratories). All washes and dilutions were performed in PBS containing 0.1% saponin. Sections were incubated with an anti-Ki-67 antibody (clone SP6, ab16668, abcam), anti-B220 antibody (clone RA3-6B2, R&D Systems, Minneapolis, MN, USA), or anti-CD3 antibody (SP7, Abcam, Cambridge, UK) containing 3% human serum overnight at room temperature. After blocking with 1% goat serum for 15 min sections were incubated with a biotin-conjugated secondary antibody (goat anti-rabbit IgG or goat anti rat IgG, Vector laboratories) containing 1% goat and 3% human serum at room temperature for 30 min. For detection of an ABC complex (Elite ABC kit, Vector laboratories) was used before the sections were developed using diaminobenzidine (DAB Peroxidase Substrate kit; Vector Laboratories) as chromogen. Sections were counterstained with Mayer´s hematoxylin (Histolab). For immunofluorescence detection of CD3-positive cells, sections were incubated with anti-CD3 antibody (SP7, Abcam, Cambridge, UK) in 4% goat serum overnight at 4 °C following incubation with secondary Goat anti-rabbit-Alexa Fluor 488 (Thermo fisher Scientific). Histological assessment of the transgenic mouse tissues was performed by a pathologist. For full list of antibodies used for immunohistological analyses see Table S3.

### 4.6. Whole-Exome Sequencing, Variant Calling, and Copy Number Alterations in Mice

Whole-exome sequencing (WES) was performed on DNA from totally 24 samples; 15 independently developed thymic lymphomas and thymus from nine controls. The controls were divided into three different groups corresponding to (a) irradiated *PPM1D*-transgenes, (b) irradiated wild-type, and (c) non-irradiated wild-type. In the respective control groups, three mice, representing three different founder strains, were subjects for sequencing.

WES was performed by Otogenetics (Otogenetics Corporation, Atlanta, GA, USA) through pair-end sequencing on Illumina platforms (Illumina, San Diego, CA, USA) after enrichment with Agilent SureSelectXT Mouse All Exon (Agilent technologies, Santa Clara, CA, USA) reaching an average coverage of 80X (range 47.5–99.2X) (Table S1). Read trimming, mapping to the mouse reference genome mm10, and variant calling were performed using CLC Genomics Workbench 5.0 software (CLC, Aahus, Denmark). Somatic calling of lymphoblastic tumors and irradiated controls was done using the combined sequence from the three non-irradiated wild-type mice as normal control. Only high quality called variants with a minimum 10% allele frequency and a total read coverage of ten were considered for further analysis. All synonymous variants or variants in non-coding regions except those affecting canonical splice sites were discarded. Remaining variants were assessed manually through the Integrative Genomics Viewer (https://software.broadinstitute.org/software/igv/ accessed on 8 September 2020) for removal of calls due to mapping artifacts or paralogs. Calling and visualization of copy number alterations was done using the software Control-Free (control-FREE Copy Number Caller) that generates rations from normalized read distribution between tumor and normal followed by visualization in a Shiny application as described previously [45].

### 4.7. RNA Sequencing of Mouse Tumors

Bulk RNA sequencing (RNA-seq) was performed on extracted RNA in a total of 24 samples; 10 controls (thymic tissue from healthy mice) and 14 thymic lymphomas. As with mouse tumor WES, controls were divided into four groups based on irradiation status and genotype: wild-type non-irradiated (*n* = 3), *PPM1D-*transgenic non-irradiated (*n* = 3), wild-type irradiated (*n* = 2), and *PPM1D-*transgenic irradiated (*n* = 2). Total RNA extraction, library preparation, and sequencing were performed by Otogenetics (Otogenetics Corporation, Atlanta, GA, USA) through paired-end sequencing on Illumina HiSeq 2500 (Illumina, San Diego, CA, USA) after mRNA purification using the TruSeq Stranded cDNA kit. Read length was 100–125 bp for all samples. 

Samples were aligned to the mm10 reference genome using STAR in 2-pass mode. Aligned reads were quantified using htseq-count and differential gene expression analysis was performed using the R/Bioconductor package DESeq2. After having converted mouse Ensembl gene IDs to human orthologs using the R package *gOrth*, gene set enrichment analysis was performed using the GSEA software from Broad institute and the Hallmark collection of gene sets from the Molecular Signatures Database, MSigDB, version 6.2.

### 4.8. Statistical Analysis

Statistical analyses were done with GraphPad Prism software (GraphPad Software, San Diego, CA, USA). The IC_50_ values (inhibitory concentration 50%) were determined from log concentrations–effect curves using non-linear regression analysis. T test was used to compare means between two groups and for comparison of three or more groups, one-way ANOVA followed by Bonferroni multiple-comparisons post-test were used. Survival analysis was examined with log-rank test and Fisher’s test was used to test significance of association between the two categories. Correlations were assessed with Pearson test/Spearman non-parametric test. *p* < 0.05 was considered significant and all tests were two-sided. Survival curves were calculated using the Kaplan–Meier method. Regarding statistical methods for genomics and transcriptomics studies, please refer to the corresponding methods section.

## 5. Conclusions

Taken together this study shows that *PPM1D* can act as an oncogenic driver inducing tumors similar to tumors induced by p53 impairments. 

## Figures and Tables

**Table 1:** List of tumors developed in irradiated *PPM1D*-transgenic mice, related to Figure 2.

Diagnosis	n	Age at Irr *^b^*	Time from Irr to Tumor	Metastatic
		(Days)	Development *^b^* (Days)	Spread *^c^*
***PPM1D*-positive mice**				
Thymic lymphoblastic lymphoma	41	Mdn: 6 (1–314)	Mdn: 196 (124–535)	Systemic
Leukemia/lymphoma	9	Mdn: 48 (4–314)	Mdn: 342 (195–482)	Systemic
Other solid tumors	22	Mdn: 20 (3–240)	Mdn: 435 (246–683)	
Papillary serous ovarian cancer	5	Mdn: 20 (4–164)	Mdn: 415 (389–598)	Splenomegaly
Lacrimal gland tumor	6	Mdn: 455 (291–614)	Mdn: 20 (4–94)	Splenomegaly
Gastrointestinal stromal tumor	1	62	463	Splenomegaly
Gastric adenocarcinoma	1	62	463	Lung, splenomegaly
Adenocarcinoma of the lung	4	Mdn: 414 (246–463)	Mdn: 34 (4–240)	
Angiosarcoma:	2			
#1		48	199	
#2		14	319	Splenomegaly
Osteosarcoma metastasis in the lung *^a^*	1	10	303	Lung, Splenomegaly
Neuroblastoma/Adrenal tumor:	2			
#1		6	683	Liver, splenomegaly
#2		6	522	
Total number of tumors	72			
**Wild-type mice**				
Thymic lymphoblastic lymphoma	4	Mdn: 16(14–87)	Mdn: 334 (220–596)	Systemic
Adenocarcinoma of the lung	1	14	437	
Lacrimal gland tumor	1	17	517	
Total number of tumors	6			

***^a^*** Presence of proliferative lymphoblastic cells in the spleen indicative of leukemia or lymphoma. ***^b^*** Age in days given as median (Mdn) and range for groups. Irr; irradiation. ***^c^*** And/or other tumor manifestations such as splenomegaly. Systemic spread implicate infiltrates of atypical lymphocytes/lymphoblasts engaging the spleen, liver, and in some cases, the kidneys. In total, 72 tumors were diagnosed in 69 PPM1D-positive mice (three mice were diagnosed as having two primary tumors each).

## Data Availability

The WES data presented in this study are openly available in European Nucleotide Archive (ENA) study accession: http://www.ebi.ac.uk/ena/data/view/PRJEB40261 accessed on 8 September 2020. RNA sequencing data from mouse thymic lymphomas are available at GEO (Gene Expression Omnibus) under the accession number GSE157769 (token: kjsfomymtluzpsl) accessed on 25 September 2020.

## Supplementary Materials

The following are available online at https://www.mdpi.com/article/10.3390/cancers13215493/s1, Figure S1: *PPM1D*/WIP1-transgenic mice demonstrate clear distinction between thymic lymphoma tissue and control tissue, Table S1: Genomic alterations detected in *PPM1D*-transgenic mouse tumors through WES, Table S2: Differential gene expression analysis on *PPM1D*-transgenic mouse tumors, Table S3: List of antibodies used in transgenic mouse tissue analysis by Western blot and immunohistochemistry. File S1: Original Western blot images of transgenic-*PPM1D* murine tissue corresponding to Figure 1B.
