# Supplementary material for: High Expression of PPM1D Induces Tumors Phenotypically Similar to TP53 Loss-of-Function Mutations in Mice
Source: Cancers (Basel). 2021 Oct 31;13(21):5493. doi: 10.3390/cancers13215493 (PMC8582939; doi:10.3390/cancers13215493)

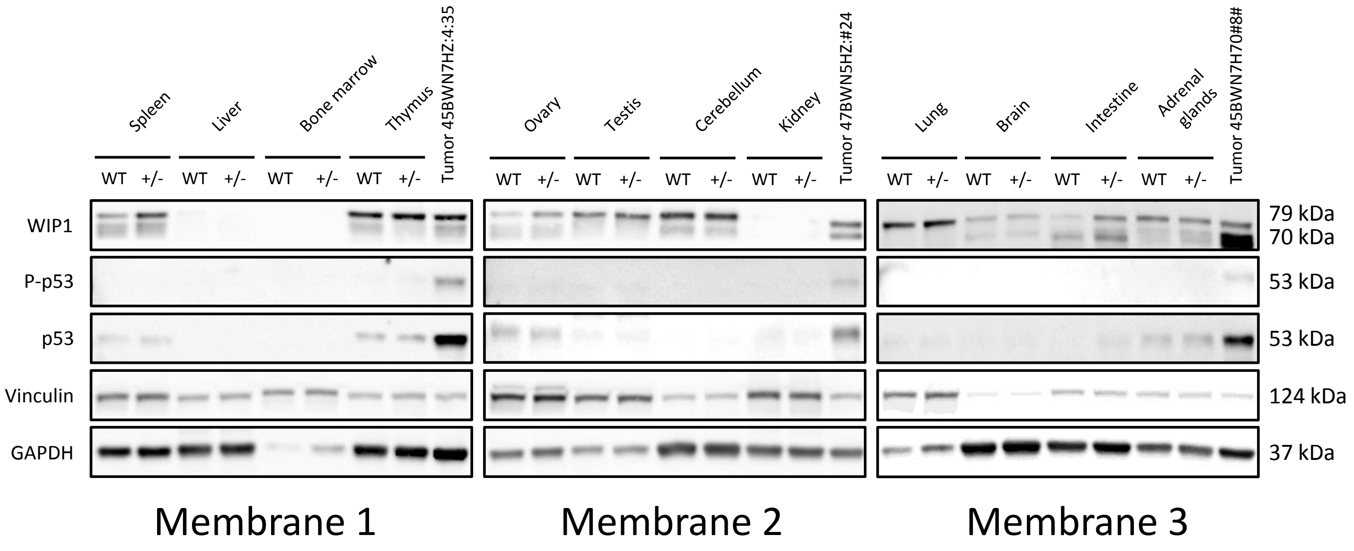

Figure X. Protein expression in mouse tissues. Protein expression level of WIP1, p53^{Ser15} phosphorylation and total p53 (rodent specific) were analyzed in wild type (WT) and heterozygous (+/-) mouse tissues. Three different tumor samples were also included. Vinculin and GAPDH levels were used as loading controls

COMPLETE MEMBRANE IMAGES FOR MOUSE TISSUES

Membrane 1: Spleen, Liver, Bone marrow, Thymus, Tumor 1
Membrane 2: Ovary, Testis, Cerebellum, Kidney, Tumor 2
Membrane 3: Lung, Brain, Intestine, Adrenal glands, Tumor 3

Membrane 1 images

WIP1 analysis including the corresponding Vinculin and GAPDH detection

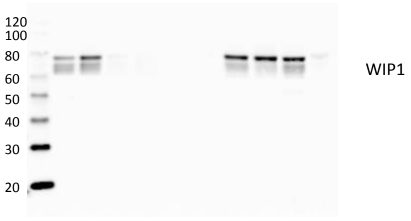

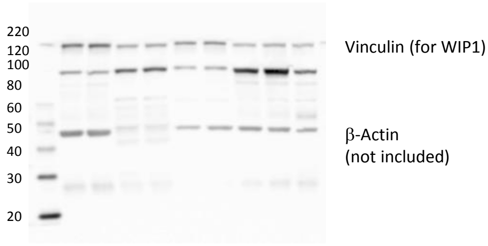

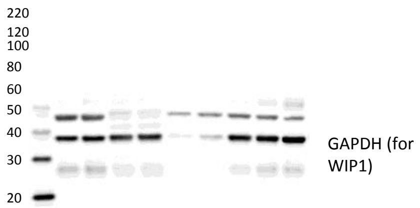

P-p53 and total p53 (rodent specific) analysis including the corresponding Vinculin and GAPDH detection

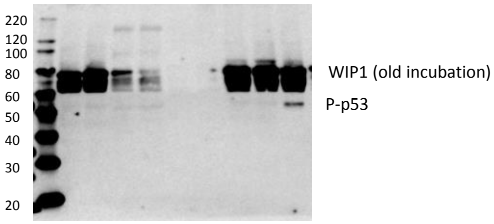

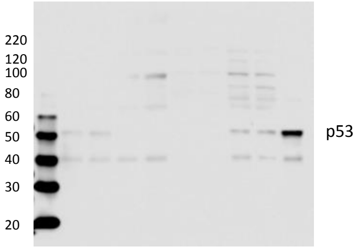

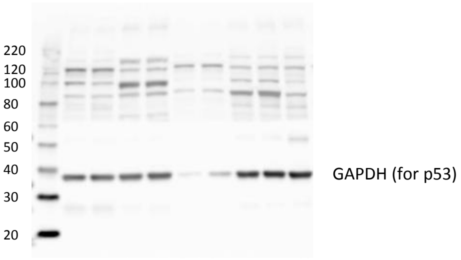

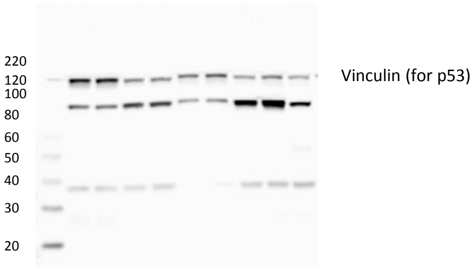

COMPLETE MEMBRANE IMAGES FOR MOUSE TISSUES

Membrane 2 images

WIP1 analysis including the corresponding Vinculin and GAPDH detection

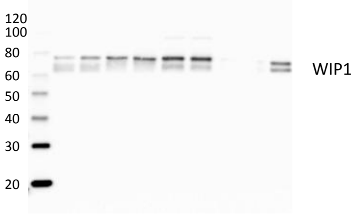

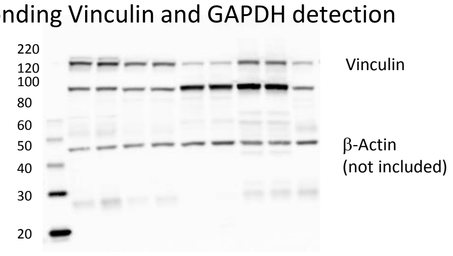

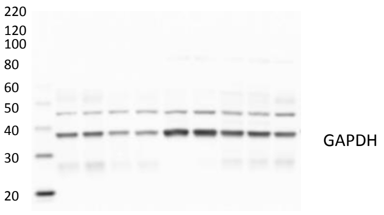

P-p53 and total p53 (rodent specific) analysis including the corresponding Vinculin and GAPDH detection

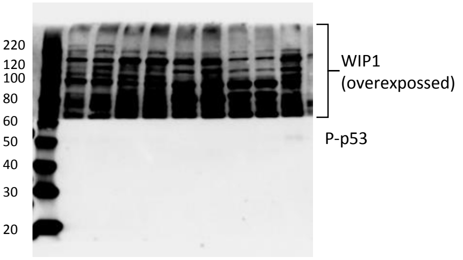

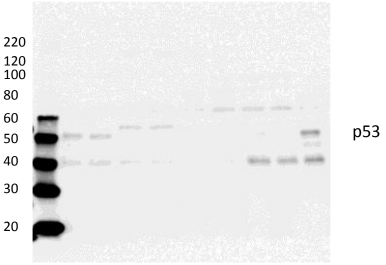

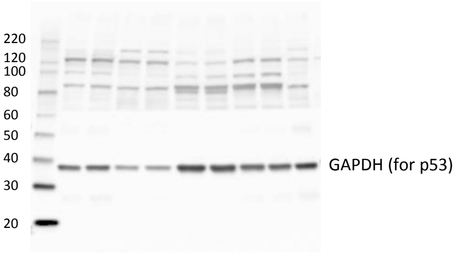

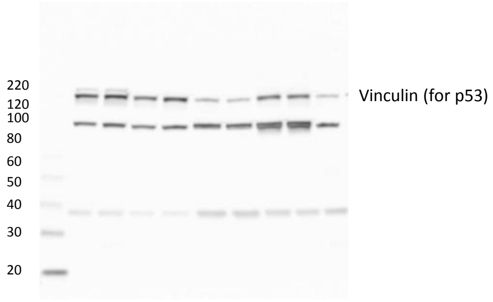

COMPLETE MEMBRANE IMAGES FOR MOUSE TISSUES

Membrane 3 images

WIP1 analysis including the corresponding Vinculin and GAPDH detection

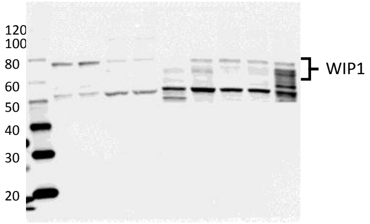

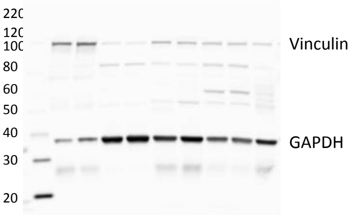

P-p53 and total p53 (rodent specific) analysis including the corresponding Vinculin and GAPDH detection

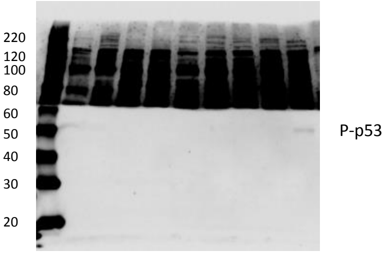

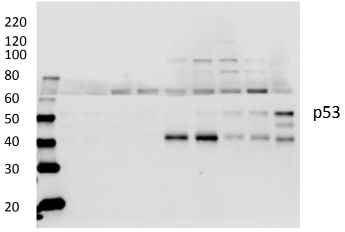

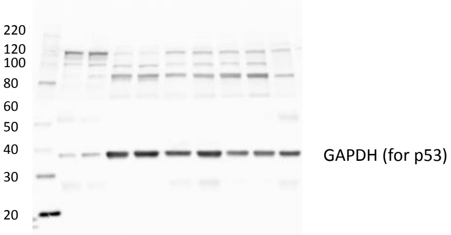

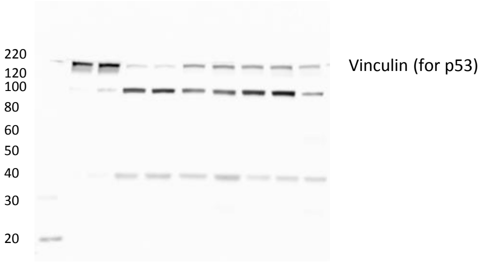

Supplement: Supplementary file 1 [file cancers-13-05493-s001.zip › Figure 1B and File S1, WB Tg-PPM1D tissues_complete membs.pdf]
