# Supplementary material for: High Expression of PPM1D Induces Tumors Phenotypically Similar to TP53 Loss-of-Function Mutations in Mice
Source: Cancers (Basel). 2021 Oct 31;13(21):5493. doi: 10.3390/cancers13215493 (PMC8582939; doi:10.3390/cancers13215493)
Supplement: Supplementary file 1 [file cancers-13-05493-s001.zip › Supp Figure S1.pdf]

Figure S1

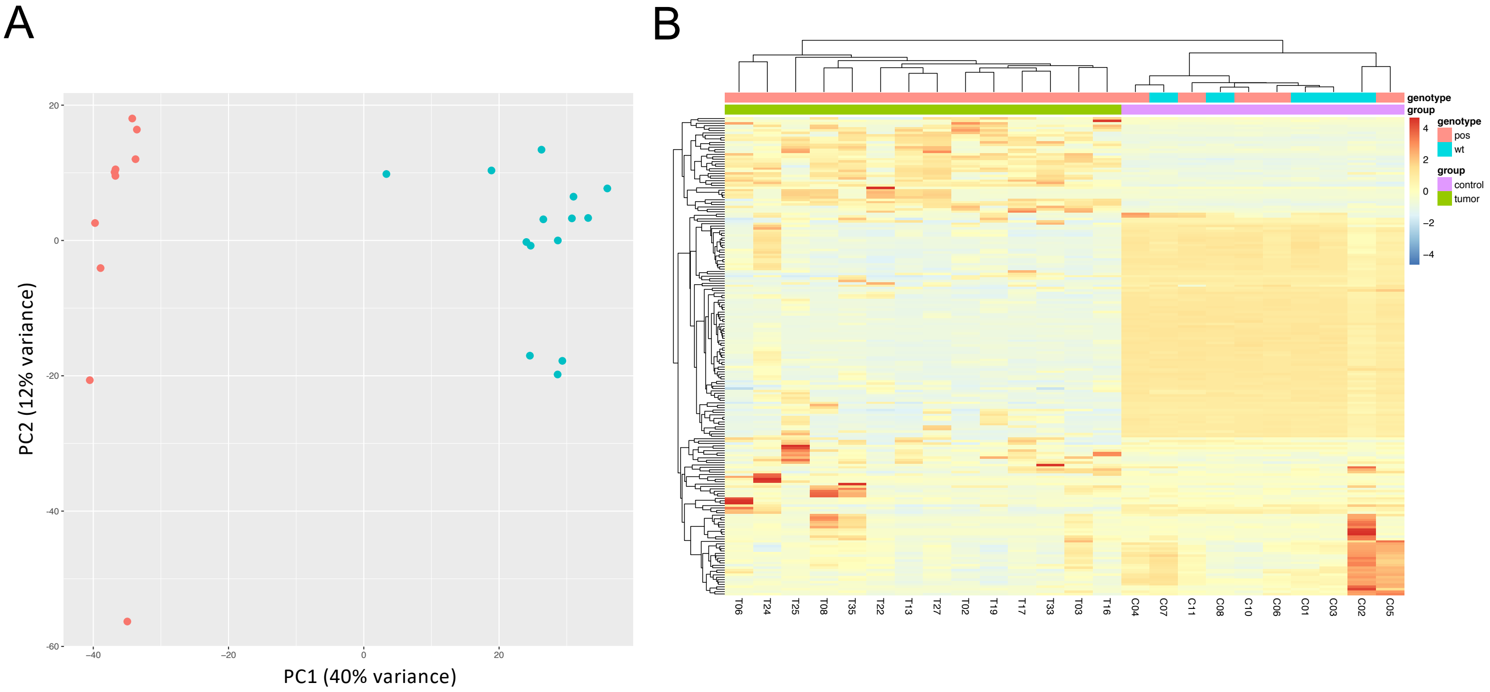

Figure S1, related to Figure 3. *PPM1D*/WIP1-transgenic mice demonstrate clear distinction between thymic lymphoma tissue and control tissue. A. Principal component analysis (PCA) plot of all samples, labelled by group (control vs tumor). Red color indicates controls; blue color indicates tumor. **B.** Unsupervised hierarchical clustering showing z scores of the 200 genes with highest standard deviation across all samples. Each column represents one sample (T = tumor; C = control). Each row represents one gene. Samples are annotated according to genotype (*PPM1D* positive or negative) and group (tumor or control). Gene expression values are scaled by row: for each gene, the mean is calculated across all sample. Each sample's deviation from this mean is then indicated by color. Red indicates higher than mean, yellow indicates similar to mean, blue indicates lower than mean.
